# White matter alterations in Parkinson’s disease with normal cognition precede grey matter atrophy

**DOI:** 10.1371/journal.pone.0187939

**Published:** 2018-01-05

**Authors:** Ivan Rektor, Alena Svátková, Lubomir Vojtíšek, Iva Zikmundová, Jirí Vaníček, András Király, Nikoletta Szabó

**Affiliations:** 1 Movement Disorders Center; First Department of Neurology, School of Medicine, Masaryk University and St. Anne’s University Hospital, Brno, Czech Republic; 2 Central European Institute of Technology (CEITEC) Masaryk University, Neuroscience Centre, Brno, Czech Republic; 3 Department of Pediatrics, University of Minnesota, Minneapolis, Minnesota, United States of America; 4 Department of Imaging, School of Medicine, Masaryk University and St. Anne’s University Hospital, Brno, Czech Republic; 5 Department of Neurology, Albert Szent-Györgyi Clinical Center, University of Szeged, Szeged, Hungary; University of Pécs Medical School, HUNGARY

## Abstract

**Introduction:**

While progressive MRI brain changes characterize advanced Parkinson’s disease (PD), little has been discovered about structural alterations in the earliest phase of the disease, i.e. in patients with motor symptoms and with normal cognition. Our study aimed to detect grey matter (GM) and white matter (WM) changes in PD patients without cognitive impairment.

**Methods:**

Twenty PD patients and twenty-one healthy controls (HC) were tested for attention, executive function, working memory, and visuospatial and language domains. High-resolution T1-weighted and 60 directional diffusion-weighted 3T MRI images were acquired. The cortical, deep GM and WM volumes and density, as well as the diffusion properties of WM, were calculated. Analyses were repeated on data flipped to the side of the disease origin.

**Results:**

PD patients did not show any significant differences from HC in cognitive functioning or in brain volumes. Decreased GM intensity was found in the left superior parietal lobe in the right (p<0.02) and left (p<0.01) flipped data. The analysis of original, un-flipped data demonstrated elevated axial diffusivity (p<0.01) in the superior and anterior corona radiata, internal capsule, and external capsule in the left hemisphere of PD relative to HC, while higher mean and radial diffusivity were discovered in the right (p<0.02 and p<0.03, respectively) and left (p<0.02 and p<0.02, respectively) in the fronto-temporal WM utilizing flipped data.

**Conclusions:**

PD patients without cognitive impairment and GM atrophy demonstrated widespread alterations of WM microstructure. Thus, WM impairment in PD might be a sensitive sign preceding the neuronal loss in associated GM regions.

## Introduction

Parkinson’s disease (PD) is associated with alterations of motor functions and a spectrum of cognitive impairments. Distinct levels of cognitive deficits are defined in PD, including mild cognitive impairment (PD-MCI) and PD dementia (PDD), although careful psychological testing could reveal a restricted deficit of certain cognitive functions even in the earliest stages of PD [1–3].

While PD-MCI and PDD are generally associated with certain level of structural impairment of both white matter (WM) and cortical grey matter (GM) (for review, see [4]), studies evaluating cognitively unimpaired PD patients have postulated contradictory conclusions. Some studies detected minor [5] or no WM changes [6]; others demonstrated extensive [7, 8] WM alterations with a critical relationship between WM changes and cognitive performance. The diversity of the results may be influenced by the fact that the studied cohorts were often defined as non-demented patients, including patients with both normal and slightly impaired cognition. We studied PD patients with no clinical cognitive impairment (PD-NCI), i.e. patients with cognition that was not significantly different from that of normal subjects. Melzer et al. [5] observed that PD with normal cognition is associated with a spatially restricted loss of microstructural WM integrity, and these alterations increase with cognitive dysfunction; Hattori et al. [6] did not observe any WM alterations in PD-NCI compared with control subjects. A similar contradiction in PD-NCI was reported for cortical alterations. A joint analysis of both GM and WM profiles in a PD cohort found extensive WM abnormalities in subjects with PD-MCI and PDD, whereas cortical atrophy was only evident in the PDD group [9]. While most studies in the PD-MCI depicted certain level of GM atrophy [10, 11], few reported absence of cortical GM atrophy in PD-MCI [12, 13], leading to the assumption that GM reduction occurs later in the disease and might be not present in PD-NCI.

Previous studies [14, 15] challenged the classical view that WM degeneration, with a loss of axons and myelin, occurs secondary to GM pathology. They also raised the intriguing possibility that WM alterations in PD might be a sensitive marker preceding the neuronal loss in associated GM regions [4]. These reports motivated our interest in WM and GM analysis in PD-NCI to answer the question of whether a WM alteration is present in early PD independent of the GM alteration. The previous studies focused on cortical GM; we studied both the cortical and subcortical (basal ganglia, thalamus) GM. To our knowledge, this is the first study comparing the hemispheric WM with the cortical as well as subcortical GM volumes.

We utilized a combined analysis of both T1-weighted and diffusion-weighted images (DWI) for a comprehensive evaluation of both GM and WM brain structures in PD-NCI. Diffusion tensor imaging (DTI) provides a comprehensive insight into WM microstructures based on the assumption that diffusion in brain tissue is not free but rather restricted by macromolecules and membranes. Fractional anisotropy (FA), as the most common DTI metric, which quantifies the degree of anisotropy, is influenced by the degree of myelination, axonal packing and size, and coherence and co-linearity of fibre organization [16]. Mean diffusivity (MD) might be affected by variances in cellularity, oedema, and necrosis; radial diffusivity (RD) reflects myelin changes; and axial diffusivity (AD) parallels axonal abnormalities. However, DTI metrics remain non-specific and the DTI outcomes should therefore be interpreted with caution [17].

Taken together, our multimodal study aimed to comprehensively test the following hypotheses:

1) cortical/subcortical GM alterations are not found or are only found in limited extents in PD-NCI; and 2) widespread WM alterations are found in PD-NCI.

If these hypotheses are correct, the WM alteration in PD would precede the major GM alterations.

## Material and methods

### Subjects

We recruited 25 PD patients from the Brno Movement Disorders Centre and 21 age- and gender-matched healthy controls (mean age: 57.9±7.24 years, age range: 41–71 years, 8 male). The patients that did not present any evident cognitive impairment at clinical examination were included to the study and were further examined utilizing detailed psychological tests. Five patients were excluded because of MRI technical problems (movement artefacts, restricted field of view, claustrophobia), drop-out rate was 20%. Twenty PD patients (mean age: 61.9±7.63; age range: 48–72 years, 11 male) that have included to the study were in the early stages of the disease with slight to moderate motor impairment (Hoehn-Yahr stage 1–1.5) and disease duration up to 5 years. In 10 patients, the right side was predominantly affected. Healthy individuals had no history of neurologic or psychiatric disease. All participants were right-handed (Table 1).

**Table 1:** Demographic data and cognitive scores.

*Demographic data*	Control	PD patients	Significance (p<)
**N (male)**	21 (8)	20 (11)	*0*.*28*
**Age (years ± SD)**	57.9 (7.24)	61.9 (7.63)	*0*.*09*
**Handedness (L/R)**	0/21	0/20	-
**Side of symptom onset (L/R)**	-	10/10	-
**Education (years ± SD)**	12.24 (3.52)	12.25 (2.92)	*0*.*99*
***Cognitive domains Z---scores***			
**Attention (range)**	---0.56---0.57	---1.85–0.46	*0*.*53*
**Executive (range)**	---1.04---0.92	---2.22---0.75	*0*.*76*
**Language (range)**	---0.95---1.69	---0.98---1.55	*0*.*94*
**Memory (range)**	---1.27---0.92	---1.33---0.75	*0*.*83*
**Visuospatial (range)**	---1.59---0.92	---1.54---0.75	*0*.*71*

The study was approved by ethics committee of St. Anne’s University Hospital. Written informed consent was obtained from all participants.

### Neuropsychological scores

In addition to a neurological examination, comprehensive neuropsychological testing *(Mattis Dementia Rating Scale (MDRS)*, *Tower of London*, *Stroop Test*, *Rey-Osterrieth Complex Figure Test*, *Wechsler Memory Scale III*, *Wechsler Adult Intelligence Scale-*, *Third Edition (WAIS III)* and *Verbal Fluency Test*) was performed by an experienced psychologist (I.Z.) on the HC group and the PD patient group after they had taken their regular medication.

For further comparisons, individual Z-scores were calculated for the separate subscales of the different tests by subtracting the predefined means from the individual raw scores and then dividing by the predefined standard deviations (*Z-score = (raw score—mean*_*STD*_*) / SD*_*STD*_). The obtained Z-scores of the selected subscales were then averaged to produce cognitive domain specific (visuospatial, memory, attention, language, and executive) Z-scores. These cognitive domain scores were used for interaction analyses to evaluate group differences between the slopes of fitted correlation lines (based on: http://core.ecu.edu/psyc/wuenschk/docs30/CompareCorrCoeff.pdf).

### MRI image acquisition

MRI scans were performed on 3T General Electric Discovery MR750 (GE Healthcare, Milwaukee, Wisconsin) using a 12-channel head coil. DWI data were acquired by using a dual spin-echo, single-shot, echo-planar sequence with 60 non-linear directions, b-value 1000 s/mm^2^, one non-diffusion weighted image (b = 0 s/mm^2^), repetition time: 9100 ms; echo time: minimum; slice thickness: 2 mm; 0 spacing, FOV = 256x256 mm; voxel size 2x2x2 mm was interpolated to 1×1×2 mm.

T1-weighted images were scanned using 3D FSPGR sequence with the following parameters:

TR = 9.78ms, TE = 4.3ms, TI = 450ms, flip angle = 12°, voxel size = 1x1x1mm.

### Image processing

For each subject, DWI images were converted from DICOM to NIFTI format with MRIcron (http://people.cas.sc.edu/rorden/mricron/index.html). MRI data were then processed with the FMRIB Software Library, version 6.0 (FSL; www.fmrib.ox.ac.uk/fsl).

Since the motor symptoms of PD usually start on one side, lateralised volumetric differences were expected. The literature of lateralised neurological diseases demonstrated importance of the flipping of the images along the x-axis for pooling the symptoms virtually to one side [18]. So, as first step flipped analyses were performed to examine connections between GM/WM alterations and the side of the symptom onset. Three different versions of the same analysis were carried out: 1) images of patients with symptoms that started on the left side were mirrored about the x-axis (as if all the patients had symptoms that started on the right side—*RightMirr*); 2) images of patients with symptoms that started on the right side were mirrored about the x-axis (as if all the patients had symptoms that started on the left side—*LeftMirr*); and 3) non-mirrored images were used (symptoms started on the left and right sided mixed—*OrigSided*).

While the flipping method can only be used if there is no laterality in the investigated parameters, previous studies showed subtle differences between the left and the right side of the brain even in healthy subjects [19, 20]. Such asymmetry can profoundly affect the results of flipping analyses and thus challenges data interpretation. For this reason, a second method (tbss_sym) was also used to investigate the normal and altered asymmetry of the white matter diffusion parameters (see details below).

#### Tract-based spatial statistics

The diffusion data were corrected for eddy currents and movement distortions using eddy_correct tool in FSL [21]. Non-brain parts were removed from all images using the Brain Extraction Tool (BET, [22]). Diffusion tensors at each voxel were fitted by the algorithm included in the Diffusion Toolbox (FDT) of the FMRIB Software Library (FSL v. 4.0, www.fmrib.ox.ac.uk/fsl; [23]). Fractional anisotropy (FA), mean diffusivity (MD), and diffusivity parallel (AD, axial, *λ1*) and perpendicular (RD, radial, *(λ2+λ3)/2*) to the principal diffusion direction were computed for the whole brain.

The Tract-Based Spatial Statistics (TBSS [24]) tool was used to perform a voxel-wise statistical analysis of the diffusion tensor maps (“*OrigSided*”). All of the subjects’ FA maps were aligned into a standard space by co-registration to the most “typical” subject, which was the best target from all FA images based on the least number of transformations to the FA of all other subjects with non-linear registration. This option was chosen in order to achieve a better alignment of WM tracts in our population. A mean FA skeleton was created, representing the cores of all tracts common to the group. Each individual’s FA data were then projected onto this skeleton and thresholded at 0.2 FA. Voxel-wise cross-subject statistics analysis was performed using 5000 permutations, testing with the Threshold-Free Cluster Enhancement (TFCE) approach [25] adjusted for age and gender.

Analyses were repeated on data flipped to the side where the symptoms were observed at first [18] (“*RightMirr*” patients, “*LeftMirr*” patients).

Symmetry in diffusion characteristics was analysed using *tbss_sym* util with age and gender as covariates on original data without flipping. Three analyses were performed: (A.) symmetry analysis was conducted in healthy controls to depict the normal asymmetry; (B.) symmetry analysis in PD groups: all PD patients (*PD_all*), patients with right side (*PD_right*) and patients with left side (*PD_left*) symptom onset. (C.) 3-group comparison: healthy controls, *PD_left* and *PD_all*. Obviously, this analysis is restricted to only those parts of the white matter tracts that are already sufficiently close to being symmetric—i.e. where there is reasonable correspondence in general tract structure between left and right in the brain.

The Johns Hopkins University white-matter atlas was used to identify the anatomical locations of altered regions.

#### Brain volumetry

Total brain volume was estimated with SIENAX [26], part of FSL. SIENAX starts by extracting brain and skull images from the single whole-head input data. Next, tissue-type segmentation with partial volume estimation is carried out [27] in order to calculate the total volume of brain tissue. The volumetric comparison was performed using the Statistical Package for Social Sciences (SPSS 22.0 for OS X, SPSS Inc., http://www.spss.com) with age and gender as covariates, corrected for multiple comparisons.

#### Voxel-based morphometry

Local GM changes were detected by an “optimised” voxel-based-style protocol (VBM) [28] using FSL [22]. After brain extraction, tissue-type segmentation was carried out by the FAST algorithm [27]. The resulting GM partial volume images were registered to a standard space (MNI152) using linear transformation [29] followed by a non-linear registration [30]. The resulting images were averaged to create a study-specific template, to which the native GM images were then non-linearly re-registered. The registered partial volume images were then corrected for local expansion or contraction by dividing by the Jacobian of the warp field. The modulated segmented images were then smoothed with an isotropic Gaussian kernel with a sigma of 3 mm. Final statistical steps with TFCE were the same as described with TBSS.

#### Volumetry of deep brain grey matter structures

We used FIRST, a model-based segmentation/registration tool for volume comparison of the subcortical structures of the two groups [31]. This approach uses deformable surface meshes specific to subcortical structures, namely the amygdala, caudate nucleus, hippocampus, pallidum, putamen, and thalamus. Given the observed intensities in a T1-weighted image, FIRST searches through linear combinations of shape modes of variation for the most probable shape instance based on learned models. We decided not to include the nucleus accumbens due to inappropriate segmentation.

## Results

### Neuropsychological scores and clinical data

Table 1 summarizes the basic demographic data and clinical and neuropsychological scores. Patients and controls were matched for age, gender, and years of education. No statistically significant group difference was found in the neuropsychological tests. Hoehn-Yahr stage, age and gender were compared for subgroups of patients (*PD_left*, *PD_right*) and no differences were found (p>0.95).

### TBSS

**Fig 1:**
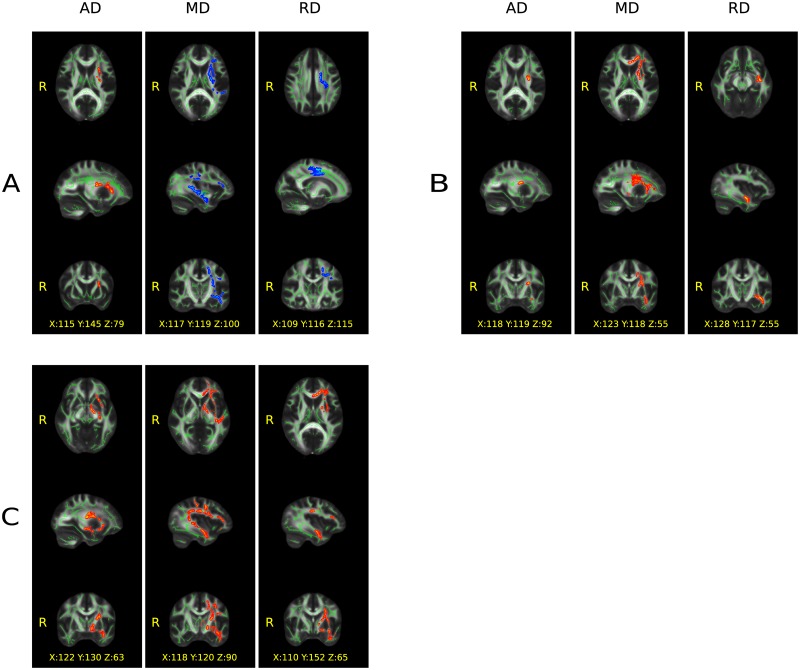
Significant group differences (p < 0.05, corrected) in the white matter revealed by tract-based spatial statistics. A) without flipping, B) after flipping to the left and C) to the right side. Color code: red = increased, blue = decreased diffusion parameters in the Parkinson patients compared to the healthy subjects; green = skeleton of the white matter tracts. AD = axial, MD = mean and RD = radial diffusion. MNI coordinates of the voxel showing the highest significance is provided on the figure.

The *OrigSided* AD in the PD group was significantly higher in the superior and anterior corona radiata, internal capsule, and external capsule on the left side than in the HC group (p<0.009, corrected). No other DTI parameter showed significant changes, although a slight trend was detected for MD and RD. Increased MD (p<0.059, corrected, in the left sided: superior corona radiata, superior longitudinal fascicle, external capsule, internal capsule, temporal WM, and prefrontal WM) and increased RD (p<0.090, corrected, in the left sided: cortico-spinal tract, superior corona radiata, body of corpus callosum, and prefrontal WM) was found in PD compared to HC (Fig 1/A). These results became significant in the *LeftMirr* (Fig 1/B) (MD: p<0.021 and RD: p<0.024, corrected) and in the *RightMirr* (Fig 1/C) (MD: p<0.016 and RD: p<0.029, corrected) comparisons. (For diffusivity parameters see S1 Fig, S1 and S2 Tables).

Hemispheric comparison in healthy individuals showed a rightward shift of AD, MD and RD dominance (p<0.05, corrected) in the frontal, parietal and temporal white matter, and a rightward shift of FA dominance along the superior longitudinal fasciculus. (The leftward shift in dominance of these parameters is the reverse of the above findings). Hemispheric comparison in PD patients revealed a slight anterior-ward shift of the above parameters, mainly affecting the secondary and primary somatosensory and primary somato-motor areas (S2 Fig). In case of the 3-group comparison (S3 Fig), in *PD_right*, the asymmetry of in RD values was detected in the left hemisphere (RD was higher in the temporal and parietal lobe), and FA was lower in the left temporal lobe compared to the controls (p<0.05, corrected). In *PD_left*, the analysis revealed a trend for the same results, but only in the temporal lobe (p<0.07, corrected). After plotting the subject-wise diffusion data under the ROIs of the 3-group comparison result (S4 Fig), the disappearing of the normal asymmetry of the RD and FA parameters could be seen in PD patients.

### Grey matter volumetry and density (SIENAX, VBM, FIRST)

No volumetric differences were found with SIENAX in GM (p<0.086, corrected) or in WM (p<0.869, corrected) volumes between PD and HC (S3 Table).

**Fig 2:**
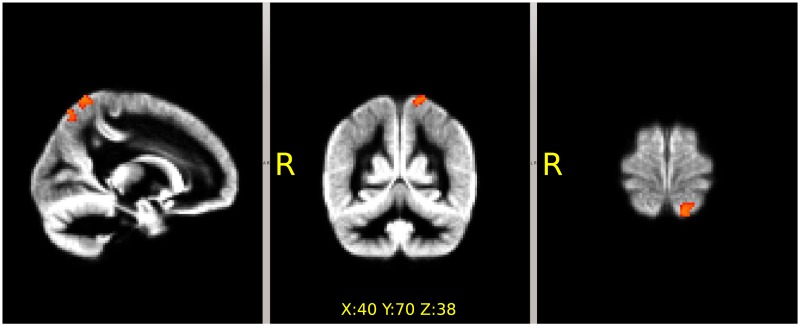
Focal decrease of the gray matter density in the Parkinson’s patients revealed by voxel-based-morphometry. The localization was the same in the comparisons with (flipped to the left side: p<0.014 and to the right: p<0.02, corrected) and without flipping (p<0.059). MNI coordinates of the voxel showing the highest significance is provided on the figure.

A tendency for decreased focal GM intensity was found with VBM in the left superior parietal lobe in *OrigSided* PD (p<0.059, corrected). It became significant in the *RightMirr* (Fig 2 (p<0.02, corrected) and (p<0.014, corrected) in the *LeftMirr* comparison.

No significant differences were found with FIRST in the volumes of subcortical GM structures between PD and HC (S5 and S6 Figs).

## Discussion

In this study, we investigated cortical/subcortical GM and WM changes in PD patients with normal cognition as compared to healthy individuals. The outcomes revealed limited differences in GM thickness in the superior parietal cortex and no change in subcortical volumes, with widespread alterations of WM. WM alterations were predominantly located in the left hemisphere.

Our results revealed early susceptibility of both GM and WM of left hemisphere in early stages PD-NCI and align with GM outcomes of Claassen and co-workers [32]. They found no influence of handedness and motor symptom asymmetry on results [32], and the current literature does not contain sufficient explanation of this phenomenon. On one hand, the analyses that included the symptom asymmetry strengthen our conclusions of critical vulnerability of the left hemisphere in early PD, since the results were more pronounced and reached higher level of significance. On the other hand, the normally existing brain asymmetry might disappear in PD due to on-going pathology in the brain, suggesting that PD might be a disconnection syndrome as Caminiti and co-workers [33] claimed in recent study. They revealed loss off functional and structural connection in PD patients’ central nerve system caused by axonal loss, which hypothesis could also support our findings.

Our findings of left-sided superior parietal atrophy are in agreement with previous studies with mild posterior atrophy already present in early PD [9, 34]. The superior parietal cortex is critically involved in attention and visual processing in healthy subjects [35] and was further investigated in PD. The posterior atrophy in the superior parietal and occipital GM was related to visual hallucinations in PD [36]. Our data showing reduced parietal thickness aligns with several studies [37–39] and contradicts others that failed to reveal any GM abnormalities in early PD [34, 40–43]. In a study by Mak et al. [40], the cortical thickness in PD-NCI did not differ from that of HC, although a subgroup that subsequently converted to PD-MCI showed temporal cortex thinning [40].

We detected no global cortical GM and focal subcortical volume change using different MRI analysis methods. Our results support the conclusions of several studies that did not detect subcortical volume changes [44–48], suggesting that volumetric alteration of deep brain GM structures may occur later in the disease course. Indeed, multiple studies described volumetric abnormalities of subcortical GM structures (e.g. striatum [49, 50], caudate nucleus [51], and thalamus [49]) in patients with advanced PD, indicating that subcortical atrophy occurs later during the disease course.

In contrast to the subtle GM atrophy, we observed a robust impairment of subcortical WM [36, 52, 53]. WM damage in PD, mostly related to cognitive decline, was reported in several studies [7, 54–56]; normal or minor changes in non-demented PD patients were also reported [5]. Limited GM alteration accompanied by widespread WM changes were reported in a few studies [9, 57]. The critical conflicts between studies might stem from differences in imaging and analysis protocol or sample size and characteristics [54].

Similarly to some of previous PD studies [54, 58, 59], but contradictory to the others [60, 61], we detected an increase in AD in PD-NCI compared to HC. AD is considered a measure of axonal consistency with higher values potentially indicating an increase of axonal co-linearity, however axonal loss in co-occurrence of inflammation [62] may provide additional explanation to elevated AD in the PD population. The activation of inflammatory processes related to activation of microglia caused by alpha-synuclein aggregation may be associated with WM abnormalities in PD [63]. Studies demonstrated decrease of AD in early stages of axonal damage [16] that is followed by pseudo-normalization of AD due to clearance of axonal and myelin debris in the WM [64] and pointed to evolving AD abnormalities during the disease on course.

While RD quantifies diffusion perpendicular to the axonal axis, with an increase indicating an abnormally low myelin content [65], the diffusion parameters indirectly reflect changes in the microstructural tissue. The elevation of RD, which quantifies diffusion perpendicular to the axonal axis and indicates an abnormally low myelin content [17], was congruently detected in various PD stages [7, 58, 59]. Theilmann et al [55] detected similar co-occurrence of elevated RD with higher AD and suggested that these changes may reflect early cell loss and gliosis in PD. It has been reported that PD is primarily caused by a synaptic dysfunction with axonal transport problems leading to subsequent cell death [66]. Also, AD and RD alterations may together contribute to increased MD that could refer to loss of tissue density [67] and it is in agreement with previously published studies [36, 52]. The pathology underlying the loss of microstructural WM integrity could be explained with immunocytochemical evidence for the presence of ubiquitin and alpha-synuclein inclusions in the axons of Lewy body disease cases, which may impair axonal transport before cell body damage [68]. The impairment of WM might underlie the disturbance of large-scale network connectivity in PD-NCI [69]. While DTI studies congruently pointed to complex PD-related WM alteration, the interpretation of actual tissue abnormalities remains challenging due to non-specificity of DTI parameters, which indirectly refer to microstructural tissue change by calculating the changes in water diffusion per se [65].

WM lesions (WML) observed in structural MRI studies have been associated with cognitive impairment in PD [70] (for review see: [71]); however, our participants were relatively young and exhibited only minor WML on anatomical images. WM changes assessed by TBSS were found in PD but not in HC, with a comparable burden of ischemic WML, indicating that the DTI-assessed WM changes reflect the initial stages of neurodegeneration [72, 73]. A recent follow-up DTI study confirmed more pronounced changes in diffusivity parameters during aging in PD than in HC [74].

Our findings in the PD-NCI cohort expand on the published works of other groups studying WM and GM integrity [40, 57, 75] by adding an analysis of subcortical GM, including the basal ganglia. Our results of nearly normal cortical GM and normal subcortical GM volume do not support the hypothesis that WM alterations reflect Wallerian degeneration and are secondary to early cortical/subcortical GM atrophy [76].

## Limitations

The possible limitations of our study should be noted. The small sample size limits power of the statistical analyses and may cause that we failed to reach statistical significance in certain measures. The diagnosis of PD lacks histopathological verification. The subjects were assessed while taking their medication, which could influence cognitive outcomes. The distinct analysis methods used in our study might have different levels of sensitivity. We cannot exclude the possibility that there were subtle changes in the GM that were not revealed by the methods we used. On the other hand, the WM impairment was robust, as in the report by Rae et al.[54]. The interpolation of DTI data makes it possible to see subtle anatomical details, although it might have potential disadvantages. While high-order interpolations may cause ringing artefacts, especially in border areas between compartments with different T2 relaxation times, no significant interpolation effects were detected on DTI indices [77]. While TBSS requires at least one diffusion-unweighted scan, averaging of multiple b0 images improves SNR and thus estimation of diffusion tensor [78]. Lower SNR of b0 scans concerns the Rician noise distribution and the presence of the rectified noise floor that underestimates mean diffusivity particularly in regions with high anisotropy [79]. However, the potential bias introduced by a limited number of b0 is common for all participants in the study, thus unlikely caused differences between patients and healthy individuals. Our findings are preliminary; future prospective longitudinal studies are needed to assess the long-term progression of WM and cortical and subcortical atrophy.

## Conclusions

Our findings confirmed widespread WM pathology in PD. Given the small extent of GM atrophy, the critical WM deficit in PD-NCI challenges the traditional view that WM degeneration, including loss of axons and myelin, occurs secondary to cortical pathology. And this WM alteration might lead to structural disconnection with the loss off normal existing brain asymmetry resulting dysfunctional brain networks. Hence, WM alterations in PD might be a sensitive preceding sign of the neuronal loss in associated GM regions [4].

## Supporting information

S1 Fig Results of TBSS analysis. Means and standard deviations are showed separately for patients and controls using box plot graphs.
(PDF)

S2 Fig Results of brain asymmetry analysis. Higher diffusivity parameters are color coded in red, lower values are color coded in blue.
(PDF)

S3 Fig Results of the three-group comparison in brain asymmetry analysis. (PDF)

S4 Fig Boxplots from the results of three-group comparison. (PDF)

S5 Fig Partial brain volumes. Volumes are in mm3, except for “VSCALE”, which stands without dimension. HC = healthy controls, PD = Parkinson patients. Red crosses refer to the outliers.
(PDF)

S6 Fig Volumes of deep brain grey matter structures. Mean values and standard deviation are shown separately for patients and controls using box plot graphs.
(PDF)

S1 Table Mean values of the diffusion parameters within the ROIs showing significant between group differences using flipping. HC = healthy controls, PD_L = Parkinson patient with symptomes starting ont he left side, PD_R = Parkinson patient with symptomes starting ont he right side. For details about OrigSide, LeftMirr and RightMirr, details of analysis are provided in the Method section of the main text.
(DOCX)

S2 Table Statistical values of the diffusion parameters under the ROIs showing significant results in group comparisons with flipping. HC = healthy controls, PD = Parkinson patients. AD = axial diffusion, MD = mean diffusion, RD = radial diffusion. Coordinates are located in MNI space. For details about OrigSide, LeftMirr and RightMirr, please see section, Methods” in the main text. A-B-C represents the letter of the associated tile in Fig 1.
(DOCX)

S3 Table Partial brain volumes (SIENAX). HC = healthy control subjects, PD = Parkinson patient. ICV = intracranial volume, Brain Total = total brain volume, GM = total gray matter volume, WM = white matter volume, pGM = peripheral gray matter (cortex) volume, vCSF = ventricular cerebro-spinal fluid volume.
(DOCX)
