# Supplementary figures and images for: White matter alterations in Parkinson’s disease with normal cognition precede grey matter atrophy
Source: PLoS One. 2018 Jan 5;13(1):e0187939. doi: 10.1371/journal.pone.0187939 (PMC5755732; doi:10.1371/journal.pone.0187939)

AD

MD

RD

OrigSide

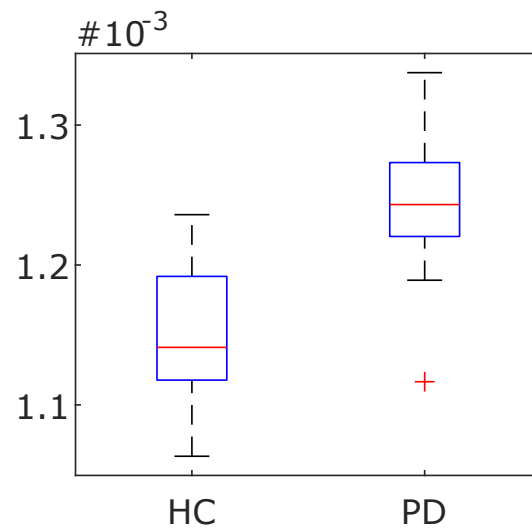

LeftMirr

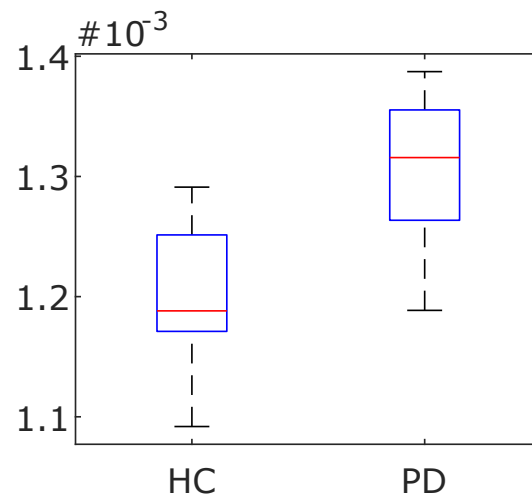

RightMirr

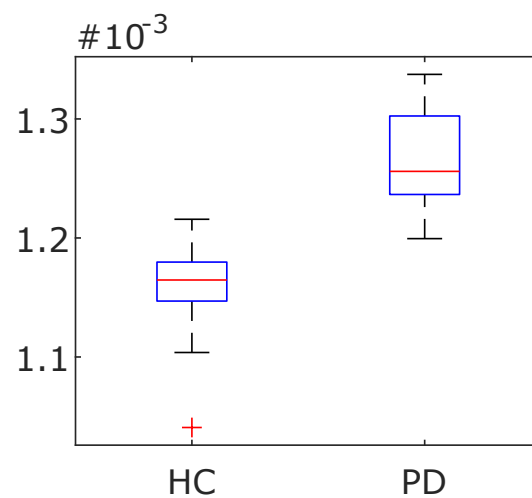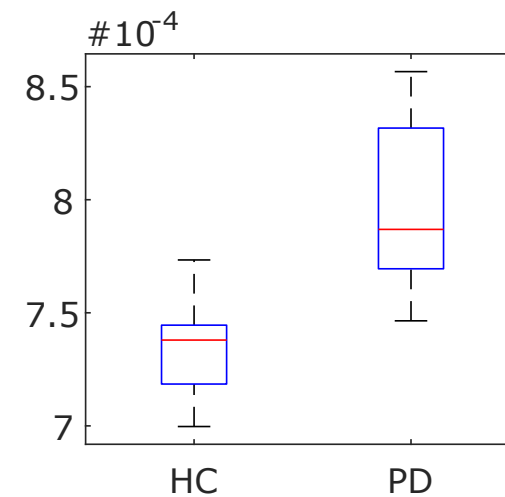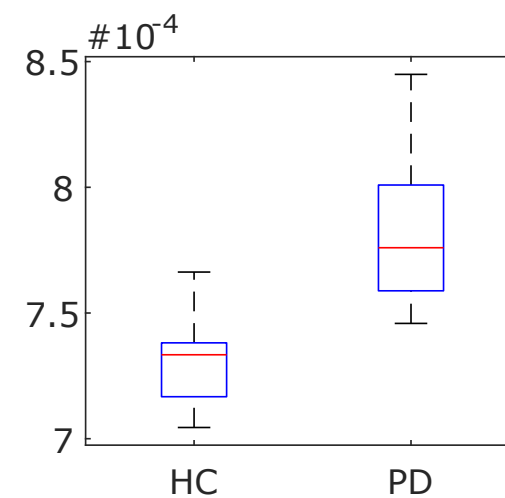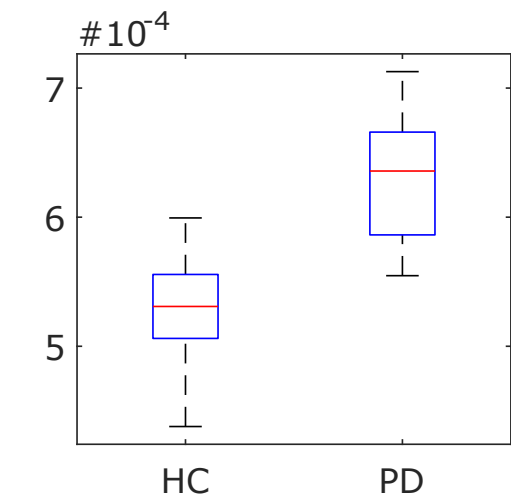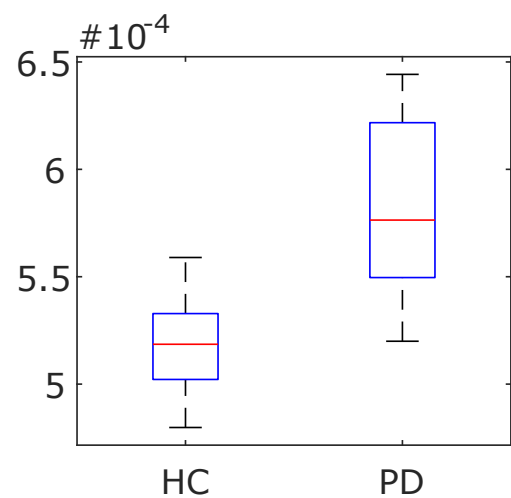

Supplement: S1 Fig — Means and standard deviations are showed separately for patients and controls using box plot graphs. (PDF) [file pone.0187939.s001.pdf]

# Comparing the left/right ratio of diffusion parameters

**PD\_right > Control (RD)**

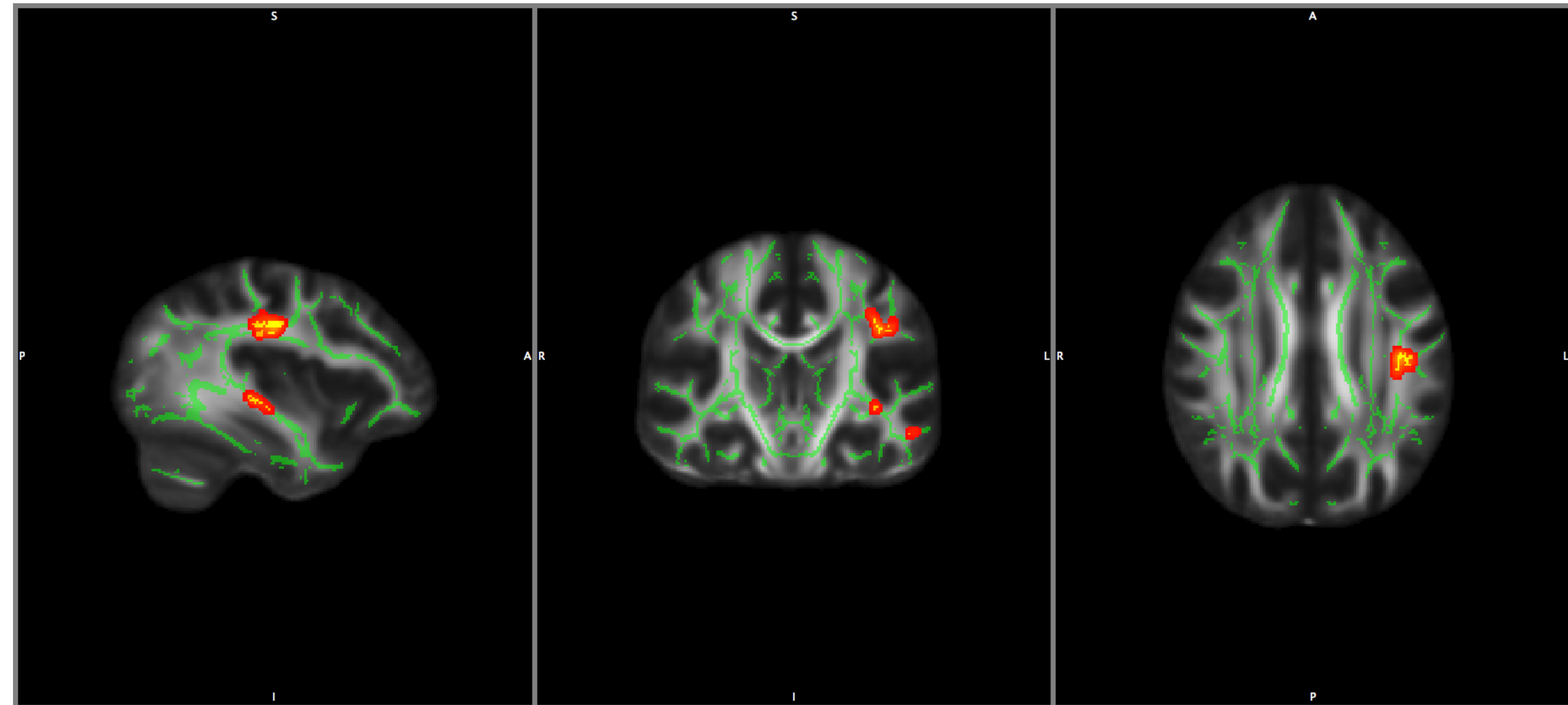

**PD\_right < Control (FA)**

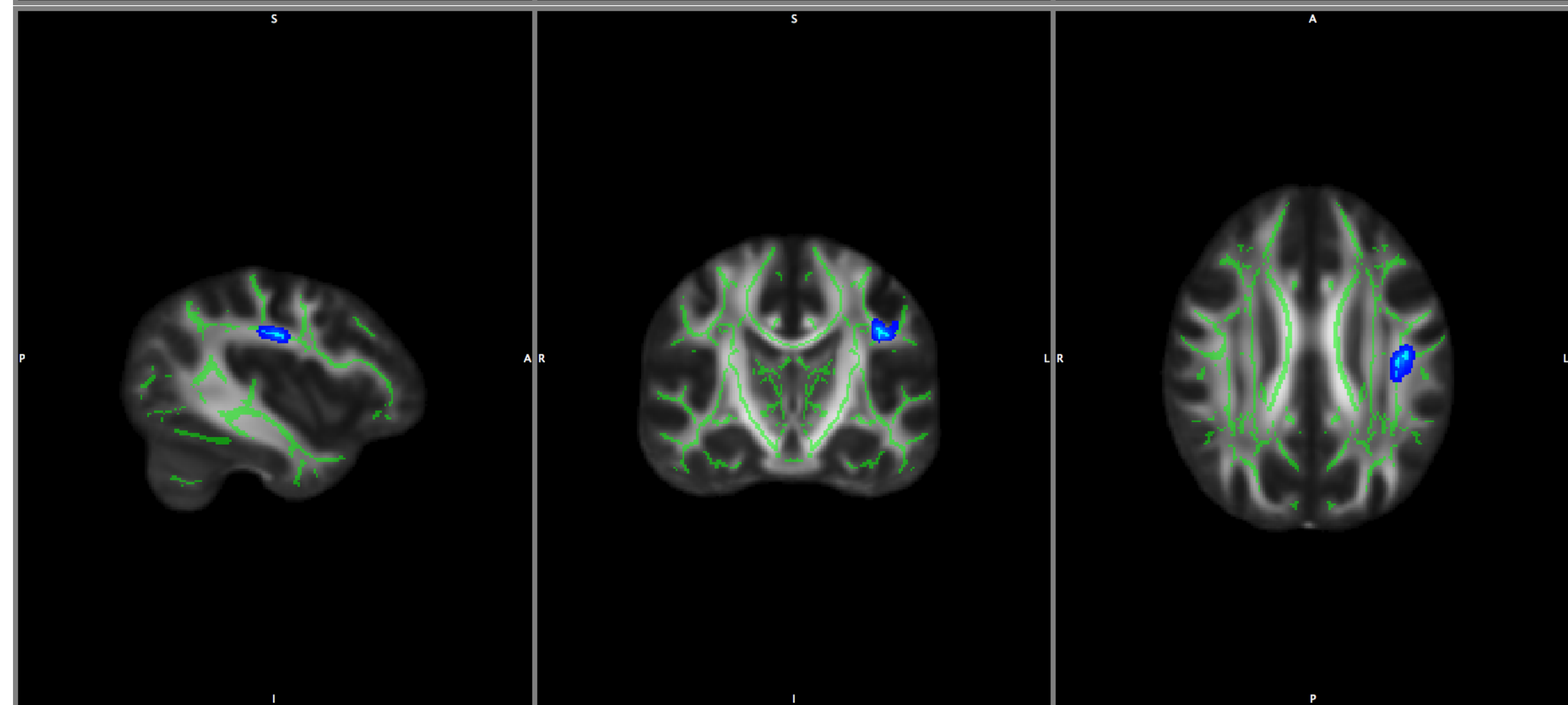

Supplement: S3 Fig — (PDF) [file pone.0187939.s003.pdf]

FA on the Left

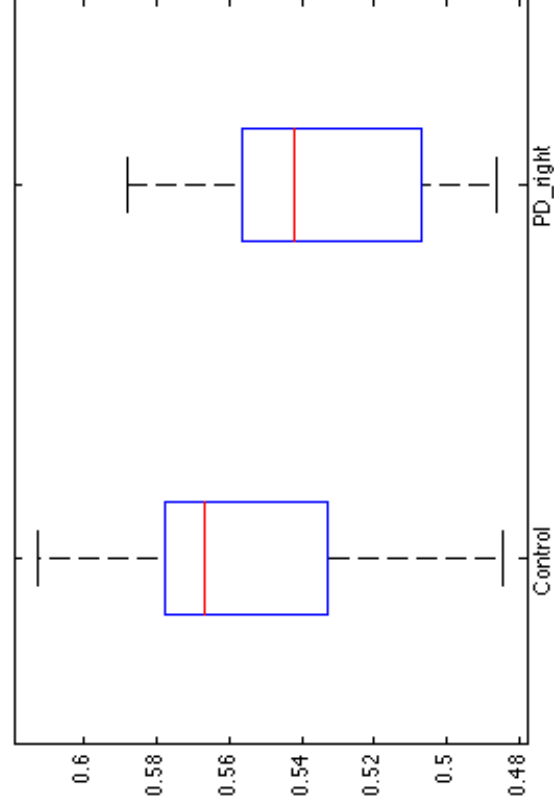

FA on the Right

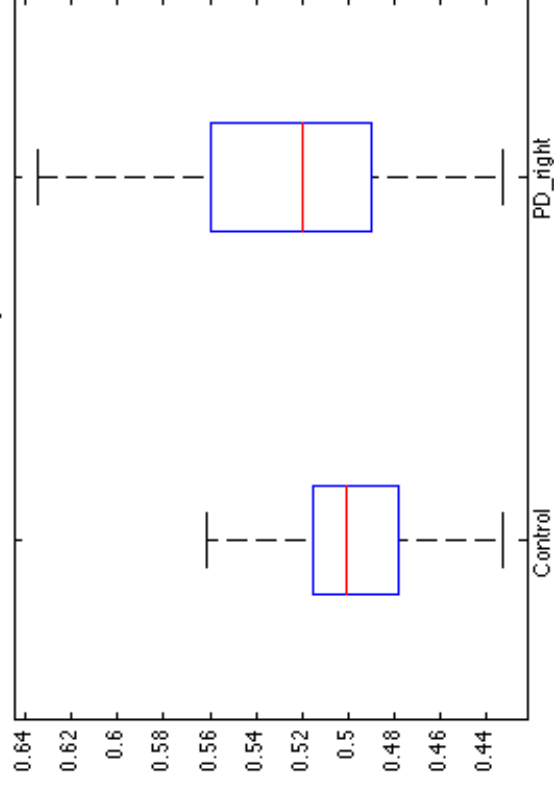

RD on the Left

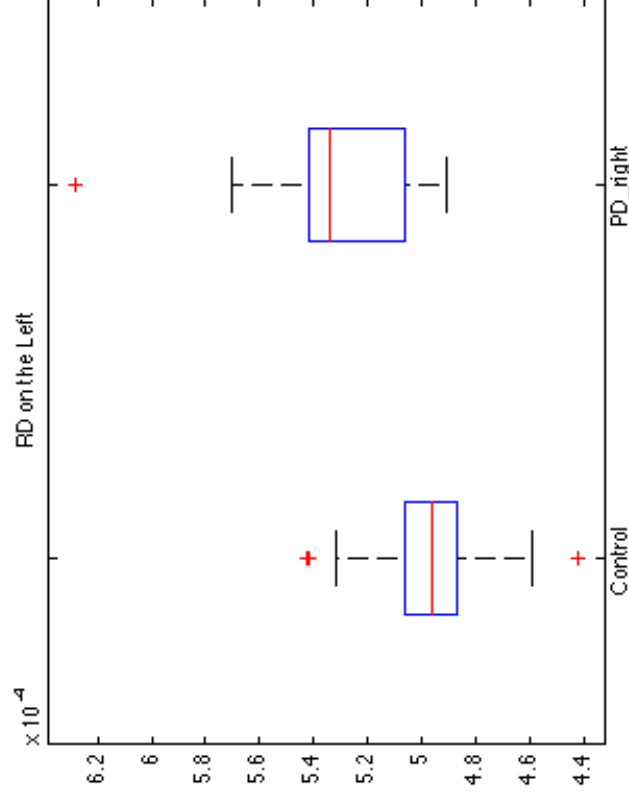

RD on the Right

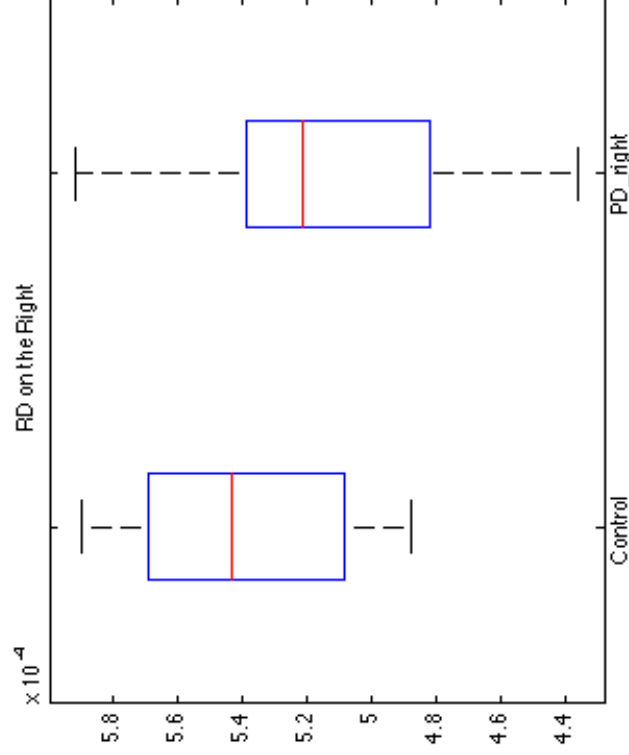

Supplement: S4 Fig — (PDF) [file pone.0187939.s004.pdf]

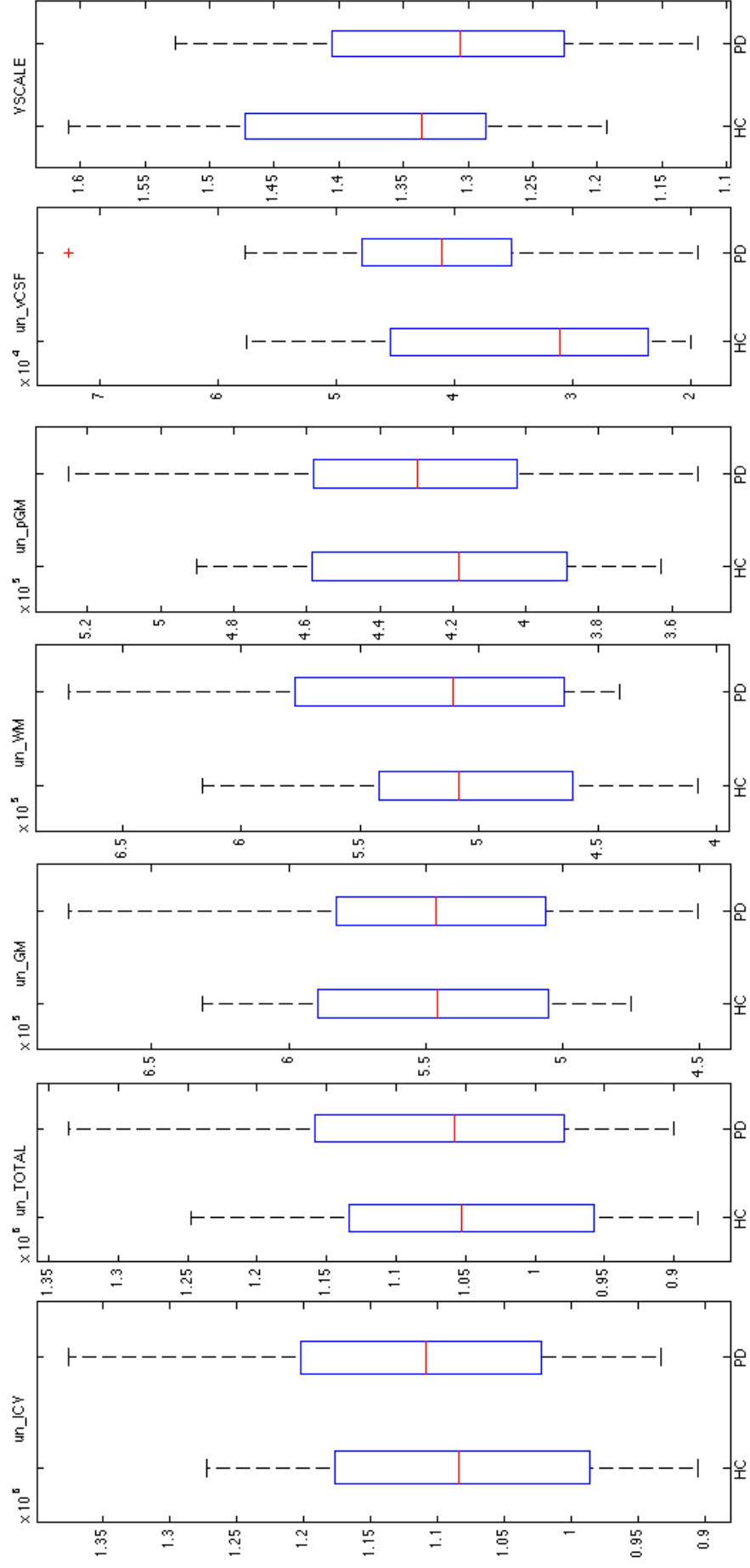

Supplement: S5 Fig — Volumes are in mm3, except for “VSCALE”, which stands without dimension. HC = healthy controls, PD = Parkinson patients. Red crosses refer to the outliers. (PDF) [file pone.0187939.s005.pdf]

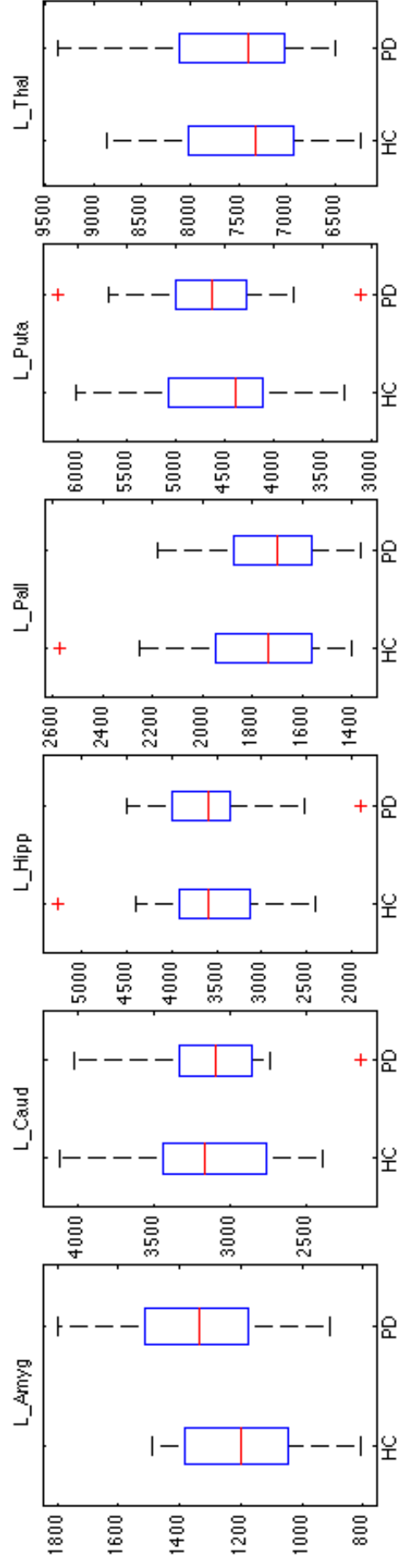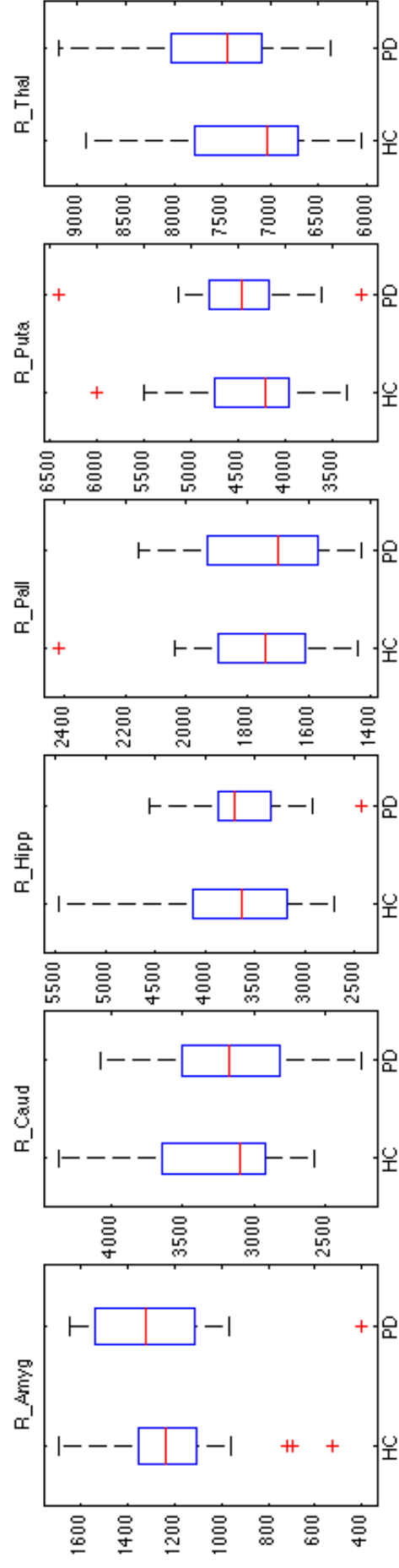

Supplement: S6 Fig — Mean values and standard deviation are shown separately for patients and controls using box plot graphs. (PDF) [file pone.0187939.s006.pdf]
