# Supplementary material for: White matter alterations in Parkinson’s disease with normal cognition precede grey matter atrophy
Source: PLoS One. 2018 Jan 5;13(1):e0187939. doi: 10.1371/journal.pone.0187939 (PMC5755732; doi:10.1371/journal.pone.0187939)
Supplement: S1 Table — HC = healthy controls, PD_L = Parkinson patient with symptomes starting ont he left side, PD_R = Parkinson patient with symptomes starting ont he right side. For details about OrigSide, LeftMirr and RightMirr, details of analysis are provided in the Method section of the main text. (DOCX) [file pone.0187939.s007.docx]

**S1 Table. Mean values of the diffusion parameters under the ROIs showing significant results in group comparisons with flipping.** HC = healthy controls, PD_L = Parkinson patient with symptomes starting ont he left side, PD_R = Parkinson patient with symptomes starting ont he right side. For details about OrigSide, LeftMirr and RightMirr, please see section „Methods” in the main text.
