# Supplementary material for: White matter alterations in Parkinson’s disease with normal cognition precede grey matter atrophy
Source: PLoS One. 2018 Jan 5;13(1):e0187939. doi: 10.1371/journal.pone.0187939 (PMC5755732; doi:10.1371/journal.pone.0187939)
Supplement: S2 Table — HC = healthy controls, PD = Parkinson patients. AD = axial diffusion, MD = mean diffusion, RD = radial diffusion. Coordinates are located in MNI space. For details about OrigSide, LeftMirr and RightMirr, please see section, Methods” in the main text. A-B-C represents the letter of the associated tile in Fig 1. (DOCX) [file pone.0187939.s008.docx]

S2 Table.
