# Supplementary material for: White matter alterations in Parkinson’s disease with normal cognition precede grey matter atrophy
Source: PLoS One. 2018 Jan 5;13(1):e0187939. doi: 10.1371/journal.pone.0187939 (PMC5755732; doi:10.1371/journal.pone.0187939)
Supplement: S3 Table — HC = healthy control subjects, PD = Parkinson patient. ICV = intracranial volume, Brain Total = total brain volume, GM = total gray matter volume, WM = white matter volume, pGM = peripheral gray matter (cortex) volume, vCSF = ventricular cerebro-spinal fluid volume. (DOCX) [file pone.0187939.s009.docx]

| \|  \|  \| **ICV** \| **Brain Total** \| **GM** \| **WM** \| **pGM** \| **vCSF** \| \| --- \| --- \| --- \| --- \| --- \| --- \| --- \| --- \| \| **HC** \| ***Subject 1*** \| 1.5790 \| 1.5502 \| 0.7685 \| 0.7817 \| 0.5795 \| 0.0289 \| \| ***Subject 2*** \| 1.4108 \| 1.3570 \| 0.7245 \| 0.6325 \| 0.5570 \| 0.0538 \| \| ***Subject 3*** \| 1.5104 \| 1.4673 \| 0.7743 \| 0.6930 \| 0.6011 \| 0.0431 \| \| ***Subject 4*** \| 1.4846 \| 1.4390 \| 0.7607 \| 0.6783 \| 0.5861 \| 0.0456 \| \| ***Subject 5*** \| 1.4908 \| 1.4437 \| 0.7577 \| 0.6860 \| 0.5915 \| 0.0471 \| \| ***Subject 6*** \| 1.4857 \| 1.4498 \| 0.7571 \| 0.6927 \| 0.5713 \| 0.0359 \| \| ***Subject 7*** \| 1.5380 \| 1.4835 \| 0.7642 \| 0.7192 \| 0.5877 \| 0.0545 \| \| ***Subject 8*** \| 1.4888 \| 1.4474 \| 0.7685 \| 0.6790 \| 0.5893 \| 0.0413 \| \| ***Subject 9*** \| 1.5123 \| 1.4399 \| 0.7458 \| 0.6941 \| 0.5702 \| 0.0724 \| \| ***Subject 10*** \| 1.3953 \| 1.3258 \| 0.6673 \| 0.6584 \| 0.5220 \| 0.0695 \| \| ***Subject 11*** \| 1.5653 \| 1.5203 \| 0.8028 \| 0.7175 \| 0.6285 \| 0.0450 \| \| ***Subject 12*** \| 1.5316 \| 1.4689 \| 0.7519 \| 0.7169 \| 0.5842 \| 0.0628 \| \| ***Subject 13*** \| 1.4895 \| 1.4583 \| 0.7461 \| 0.7122 \| 0.5742 \| 0.0312 \| \| ***Subject 14*** \| 1.4258 \| 1.3930 \| 0.7177 \| 0.6753 \| 0.5587 \| 0.0328 \| \| ***Subject 15*** \| 1.4335 \| 1.3925 \| 0.7245 \| 0.6681 \| 0.5684 \| 0.0410 \| \| ***Subject 16*** \| 1.5153 \| 1.4384 \| 0.7361 \| 0.7023 \| 0.5916 \| 0.0769 \| \| ***Subject 17*** \| 1.4876 \| 1.4251 \| 0.7173 \| 0.7079 \| 0.5637 \| 0.0625 \| \| ***Subject 18*** \| 1.3258 \| 1.2923 \| 0.6952 \| 0.5970 \| 0.5461 \| 0.0336 \| \| ***Subject 19*** \| 1.4669 \| 1.4304 \| 0.7773 \| 0.6531 \| 0.5965 \| 0.0365 \| \| ***Subject 20*** \| 1.4804 \| 1.4459 \| 0.7804 \| 0.6655 \| 0.6130 \| 0.0344 \| \| ***Subject 21*** \| 1.5526 \| 1.5233 \| 0.7710 \| 0.7523 \| 0.5983 \| 0.0293 \| \| **PD** \| ***Subject 1*** \| 1.5428 \| 1.4985 \| 0.7633 \| 0.7352 \| 0.5892 \| 0.0443 \| \| ***Subject 2*** \| 1.2879 \| 1.2321 \| 0.6162 \| 0.6158 \| 0.5170 \| 0.0558 \| \| ***Subject 3*** \| 1.4491 \| 1.3714 \| 0.7086 \| 0.6628 \| 0.5590 \| 0.0777 \| \| ***Subject 4*** \| 1.4549 \| 1.4093 \| 0.7510 \| 0.6583 \| 0.5909 \| 0.0456 \| \| ***Subject 5*** \| 1.4021 \| 1.3444 \| 0.7045 \| 0.6399 \| 0.5365 \| 0.0577 \| \| ***Subject 6*** \| 1.4256 \| 1.3772 \| 0.7005 \| 0.6768 \| 0.5386 \| 0.0483 \| \| ***Subject 7*** \| 1.5374 \| 1.4300 \| 0.7410 \| 0.6890 \| 0.6046 \| 0.1074 \| \| ***Subject 8*** \| 1.3647 \| 1.2950 \| 0.6734 \| 0.6216 \| 0.5242 \| 0.0697 \| \| ***Subject 9*** \| 1.4585 \| 1.3894 \| 0.7197 \| 0.6697 \| 0.5899 \| 0.0691 \| \| ***Subject 10*** \| 1.4671 \| 1.4090 \| 0.7181 \| 0.6910 \| 0.5609 \| 0.0580 \| \| ***Subject 11*** \| 1.5780 \| 1.5243 \| 0.7719 \| 0.7524 \| 0.5963 \| 0.0536 \| \| ***Subject 12*** \| 1.5014 \| 1.4397 \| 0.7042 \| 0.7356 \| 0.5281 \| 0.0617 \| \| ***Subject 13*** \| 1.5196 \| 1.4748 \| 0.7876 \| 0.6872 \| 0.6042 \| 0.0448 \| \| ***Subject 14*** \| 1.4898 \| 1.4614 \| 0.7840 \| 0.6773 \| 0.6092 \| 0.0284 \| \| ***Subject 15*** \| 1.4989 \| 1.4523 \| 0.7073 \| 0.7450 \| 0.5598 \| 0.0467 \| \| ***Subject 16*** \| 1.3917 \| 1.3373 \| 0.6882 \| 0.6491 \| 0.5442 \| 0.0544 \| \| ***Subject 17*** \| 1.4601 \| 1.4048 \| 0.6984 \| 0.7063 \| 0.5363 \| 0.0553 \| \| ***Subject 18*** \| 1.3911 \| 1.3392 \| 0.6744 \| 0.6648 \| 0.5170 \| 0.0519 \| \| ***Subject 19*** \| 1.6194 \| 1.5795 \| 0.7706 \| 0.8089 \| 0.5984 \| 0.0399 \| \| ***Subject 20*** \| 1.4916 \| 1.4420 \| 0.7471 \| 0.6949 \| 0.5883 \| 0.0495 \| \|  \|  \| *10e6 mm^3^ \| \| \| \| \| \| |
| --- | --- | --- | --- | --- | --- | --- | --- | --- | --- | --- | --- | --- | --- | --- | --- | --- | --- | --- | --- | --- | --- | --- | --- | --- | --- | --- | --- | --- | --- | --- | --- | --- | --- | --- | --- | --- | --- | --- | --- | --- | --- | --- | --- | --- | --- | --- | --- | --- | --- | --- | --- | --- | --- | --- | --- | --- | --- | --- | --- | --- | --- | --- | --- | --- | --- | --- | --- | --- | --- | --- | --- | --- | --- | --- | --- | --- | --- | --- | --- | --- | --- | --- | --- | --- | --- | --- | --- | --- | --- | --- | --- | --- | --- | --- | --- | --- | --- | --- | --- | --- | --- | --- | --- | --- | --- | --- | --- | --- | --- | --- | --- | --- | --- | --- | --- | --- | --- | --- | --- | --- | --- | --- | --- | --- | --- | --- | --- | --- | --- | --- | --- | --- | --- | --- | --- | --- | --- | --- | --- | --- | --- | --- | --- | --- | --- | --- | --- | --- | --- | --- | --- | --- | --- | --- | --- | --- | --- | --- | --- | --- | --- | --- | --- | --- | --- | --- | --- | --- | --- | --- | --- | --- | --- | --- | --- | --- | --- | --- | --- | --- | --- | --- | --- | --- | --- | --- | --- | --- | --- | --- | --- | --- | --- | --- | --- | --- | --- | --- | --- | --- | --- | --- | --- | --- | --- | --- | --- | --- | --- | --- | --- | --- | --- | --- | --- | --- | --- | --- | --- | --- | --- | --- | --- | --- | --- | --- | --- | --- | --- | --- | --- | --- | --- | --- | --- | --- | --- | --- | --- | --- | --- | --- | --- | --- | --- | --- | --- | --- | --- | --- | --- | --- | --- | --- | --- | --- | --- | --- | --- | --- | --- | --- | --- | --- | --- | --- | --- | --- | --- | --- | --- | --- | --- | --- | --- | --- | --- | --- | --- | --- | --- | --- | --- | --- | --- | --- | --- | --- | --- | --- | --- | --- | --- | --- | --- | --- | --- | --- | --- | --- | --- | --- | --- | --- | --- |

S3 Table.
