# Supplementary material for: White matter alterations in Parkinson’s disease with normal cognition precede grey matter atrophy
Source: PLoS One. 2018 Jan 5;13(1):e0187939. doi: 10.1371/journal.pone.0187939 (PMC5755732; doi:10.1371/journal.pone.0187939)
Supplement: S2 Fig — Higher diffusivity parameters are color coded in red, lower values are color coded in blue. (PDF) [file pone.0187939.s002.pdf]

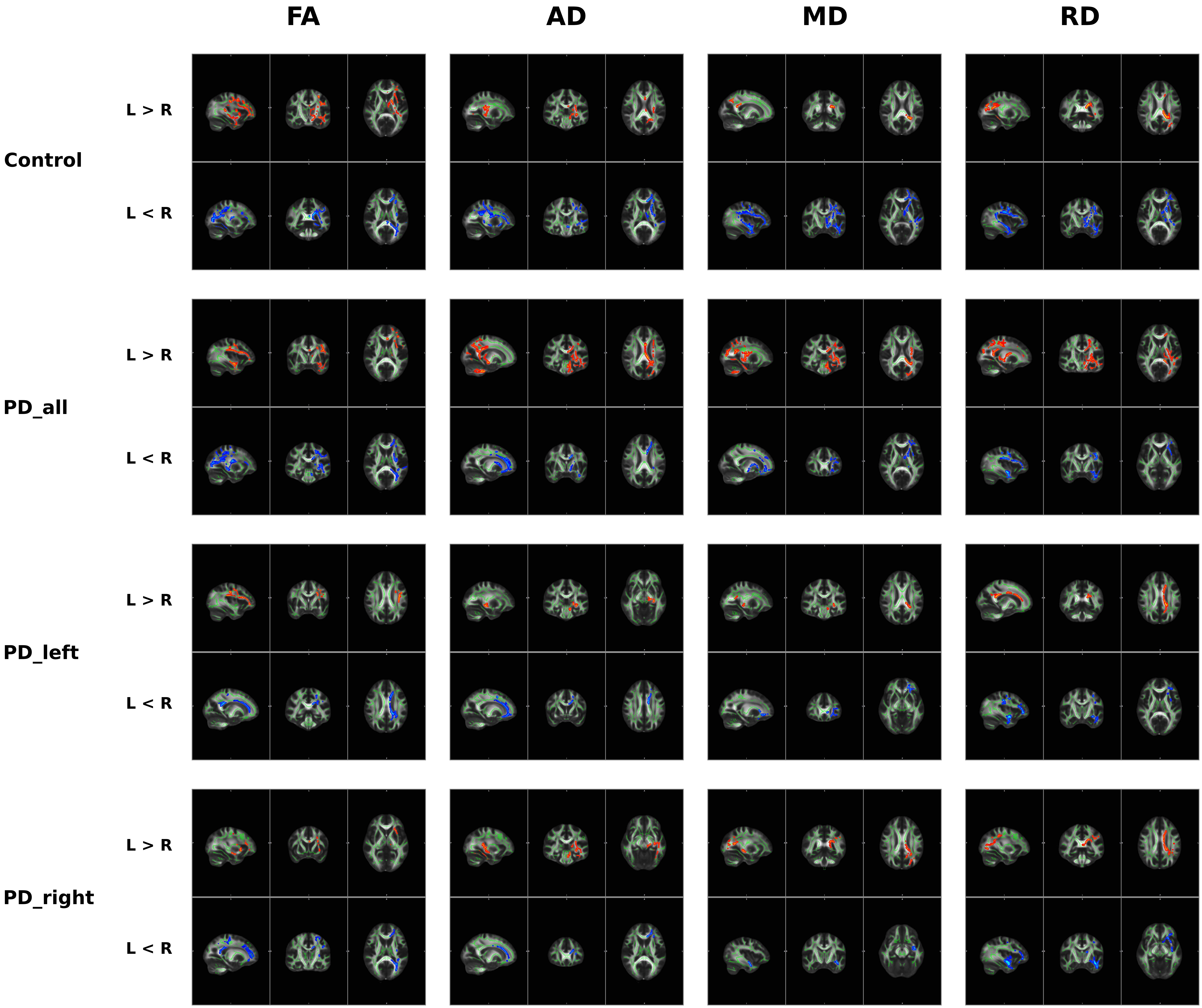
FA
AD
MD
RD
Control
PD_all
PD_left
PD_right
L > R
L < R
